# Phenotypic and genotypic characterization of *Stenotrophomonas maltophilia *isolates from patients with cystic fibrosis: Genome diversity, biofilm formation, and virulence

**DOI:** 10.1186/1471-2180-11-159

**Published:** 2011-07-05

**Authors:** Arianna Pompilio, Stefano Pomponio, Valentina Crocetta, Giovanni Gherardi, Fabio Verginelli, Ersilia Fiscarelli, Giordano Dicuonzo, Vincenzo Savini, Domenico D'Antonio, Giovanni Di Bonaventura

**Affiliations:** 1 Center of Excellence on Aging, "G. d'Annunzio" University Foundation, Via Colle dell'Ara, Chieti, 66100, Italy; 2 Department of Biomedical Sciences, "G. d'Annunzio" University of Chieti-Pescara, Via dei Vestini 31, Chieti, 66100, Italy; 3 Center for Integrated Research, "Campus Biomedico" University, Via Alvaro del Portillo 21, Rome, 00128, Italy; 4 Department of Oncology and Neurosciences, "G. d'Annunzio" University of Chieti-Pescara, Via dei Vestini 31, Chieti, 66100, Italy; 5 "Bambino Gesù" Children's Hospital and Research In stitute, P.zza Sant'Onofrio 4, Rome, 00165, Italy; 6 Department of Transfusional Medicine, "Spirito Santo" Hospital, Via Fonte Romana, Pescara, 65100, Italy

## Abstract

**Background:**

*Stenotrophomonas maltophilia *is emerging as one of the most frequently found bacteria in cystic fibrosis (CF) patients. In the present study, phenotypic and genotypic traits of a set of 98 isolates of *S. maltophilia *obtained from clinical (CF and non-CF patients) and environmental sources were comparatively evaluated.

**Results:**

*S. maltophilia *exhibited a high level of genomic diversity in both CF and non-CF group, thus possibly allowing this bacterium to expand its pathogenic potentials. Strains sharing the same pulsotype infected different patients, thus likely indicating the occurrence of clonal spread or acquisition by a common source. CF isolates differed greatly in some phenotypic traits among each other and also when compared with non-CF isolates, demonstrating increased mean generation time and susceptibility to oxidative stress, but reduced ability in forming biofilm. Furthermore, in CF isolates flagella- and type IV pili-based motilities were critical for biofilm development, although not required for its initiation. Sequential isogenic strains isolated from the same CF patient displayed heterogeneity in biofilm and other phenotypic traits during the course of chronic infection. CF and non-CF isolates showed comparable virulence in a mouse model of lung infection.

**Conclusions:**

Overall, the phenotypic differences observed between CF and non-CF isolates may imply different selective conditions and persistence (adaptation) mechanisms in a hostile and heterogeneous environment such as CF lung. Molecular elucidation of these mechanisms will be essential to better understand the selective adaptation in CF airways in order to design improved strategies useful to counteract and eradicate *S. maltophilia *infection.

## Background

*Stenotrophomonas maltophilia *is a Gram-negative opportunistic pathogen in hospitalized or compromised patients [1,2]. In the last decade, it has emerged as one of the most frequently found bacteria in cystic fibrosis (CF) patients [3,4]. However, the role of this opportunistic pathogen as an innocent bystander or causative agent often remains unclear [5,6] and little is known about its virulence factors [7-9].

Biofilms, sessile structured bacterial communities exhibiting recalcitrance to antimicrobial compounds and persistence despite sustained host defenses, are increasingly recognized as a contributing factor to disease pathogenesis in CF and other respiratory tract diseases associated with chronic bacterial infections [10,11]. While *S. maltophilia*

CF isolates are known to have the ability to form biofilms on both abiotic surfaces [12-16] and CF-derived epithelial monolayer [17], it is not clear whether there is an intrinsic difference in biofilm formation among genomically diverse environmental and clinical isolates of *S. maltophilia*.

The molecular mechanisms underlying biofilm formation in *S. maltophilia *have not been extensively studied. Recently, mutants for the glucose-1-phosphate thymidyltransferase *rmlA *gene and for the cis-11-methyl-2-dodecenoic acid *rpfF *gene are reported to decrease biofilm formation [18,19]. Further, the *spgM *gene, encoding a bifunctional enzyme with both phosphoglucomutase (PGM) and phosphomannomutase activities, could be involved in biofilm-forming ability because of the homology with the *algC *gene that is responsible for the production of a PGM associated with LPS and alginate biosynthesis in *P. aeruginosa *[20].

Several typing schemes have been used successfully in the molecular epidemiology of *S. maltophilia *strains in an attempt to investigate the epidemiology of infections and nosocomial outbreaks caused by this microorganism. Phenotypic methods - such as serotyping, antibiotyping and biotyping - have proven to be poorly discriminative because of a low interstrain variability [21]. Molecular typing techniques have been successfully used to study the epidemiology of *S. maltophilia *revealing a genetically high diversity in this species [21-26].

In this study, we examined a set of 98 isolates of *S. maltophilia *- obtained from clinical (CF and non-CF patients) and environmental sources - for phenotypic (biofilm formation, mean generation time, swimming and twitching motilities, susceptibility to oxidative stress) and genotypic (clonal relatedness) traits in order to find significant differences among the groups considered. In addition, the relationship between biofilm production and the detection of *rmlA*, *spgM*, and *rpfF *genes was evaluated. Virulence was also assessed by using an experimental model of airborne lung infection.

Our results indicate that CF *S. maltophilia *isolates significantly differ in many phenotypic aspects when compared with non-CF isolates, thus suggesting the existence of a "CF phenotype".

## Results

### CF and non-CF isolates exhibit comparable relevant genetic heterogeneity

**Figure 1:**
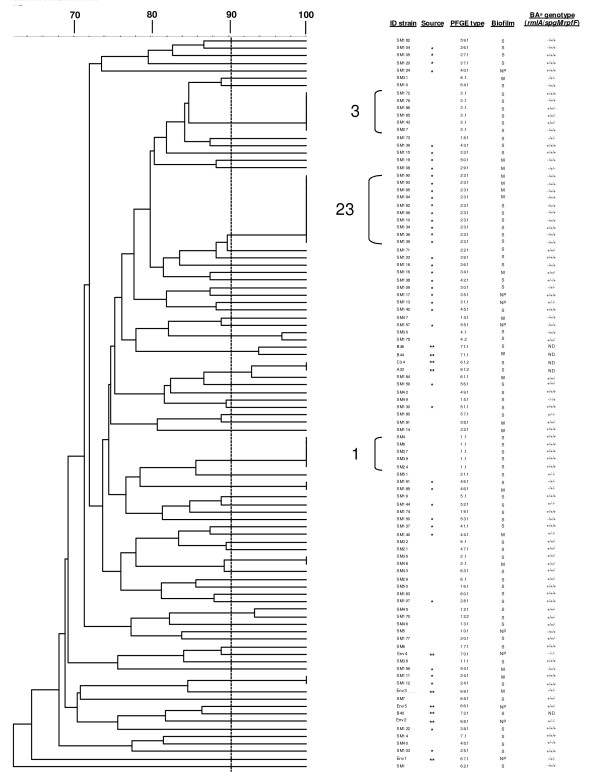
**Clonal relatedness, biofilm formation, and biofilm-associated genotypes of clinical and environmental *S. maltophilia *strains**. The dendrogram was constructed with PFGE profiles by similarity and clustering analysis by the Dice coefficient and the UPGMA. A percent genetic similarity scale is showed above the dendrogram. Isolates showing ≥ 90% of similarity (indicated as a dotted line) were considered genetically related. ID strains, source [non-CF strains are not marked, CF isolates are marked with an asterisk (*), and ENV isolates are indicated with two asterisks (**)], PFGE types and the 3 major PFGE clusters encountered in this study are also indicated. Sm189, Sm190, Sm191, Sm192, Sm193, Sm194, and Sm195 isolates were recovered from the same CF patient. Sm134, Sm135, and Sm136 strains are other consecutive isolates recovered from another CF patient. According to biofilm amount formed, strains were classified as follows: NP (no biofilm producer: OD_492 _≤ 0.096), W (weak biofilm producer: 0.096 < OD_492 _≤ 0.192), M (moderate biofilm producer: 0.192 < OD_492 _≤ 0.384), S (strong biofilm producer: OD_492 _> 0.384). ^a ^BA genotype, Biofilm-associated genotype. ND, not determined.

As shown in Figure 1, a total of 65 distinct Pulsed-Field Gel Electrophoresis **(**PFGE) types were identified among the 88 *S. maltophilia *clinical isolates studied: 36 and 29 different PFGE profiles were respectively observed among non-CF and CF isolates, showing a comparable genetic heterogeneity (number of pulsotypes/number of strains tested: 76.6 vs 70.7%, respectively; *p *> 0.05). No cases of PFGE types shared by CF and non-CF isolates were found. Eight PFGE types were represented by multiple isolates, 5 of which detected among non-CF isolates and 3 among CF isolates.

PFGE of 7 sequential isolates (Sm189, Sm190, Sm191, Sm192, Sm193, Sm194, and Sm195), collected from the same CF patient over a period of 5 years, showed the presence of two different pulsotypes (PFGE types 23.1 and 46.1). Another case of isolates recovered from the same patient was represented by isolates Sm134, Sm135, and Sm136, all sharing PFGE type 23.1. Along with visual interpretation, computer-assisted cluster analysis by using the Unweighted Pair Group Method with Arithmetic Averages (UPGMA) was also performed. Genetically related isolates showed a similarity of > 90% which corresponded to up to 3 bands of difference between 2 given PFGE profiles. Among 10 ENV isolates included in this study, 8 different PFGE types were found, with two isolates (C34, A33) sharing genetically related PFGE type with a non-CF isolate (Sm184).

### CF isolates are less effective than non-CF ones in forming biofilm

**Figure 2:**
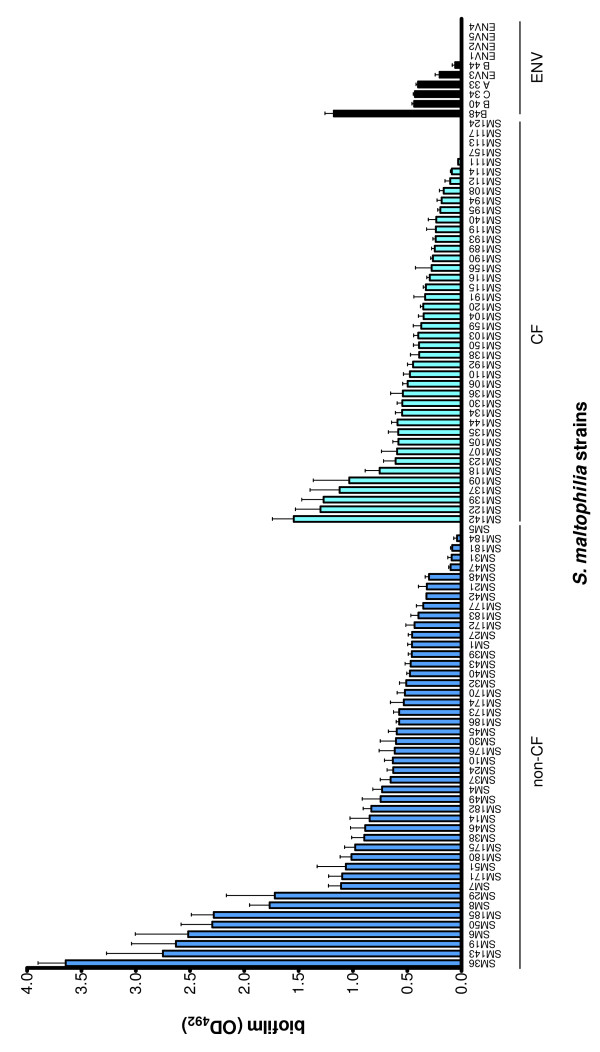
**Biofilm formed on polystyrene by 98 clinical and environmental *S. maltophilia *strains**. Biofilm amount formed after 24 h incubation at 37°C was assessed by microtiter colorimetric assay. Strains from non-CF patients are represented by blue bars, strains from CF patients are represented by cyan bars, and strains from environmental sources (ENV) are represented by black bars. Each strain was tested in quadruplicate on two different occasions. Results were subtracted from negative control (OD_492 _= 0.096) and expressed as means + SDs.

Most of *S. maltophilia *strains were able to form biofilm, although a significantly higher proportion of biofilm-positive strains was observed among non-CF strains, compared to CF ones (97.9 vs 90.2%, respectively; *p *= 0.03) (Figure 2).

Biofilm forming ability varied greatly among strains tested (OD_492 _range: 0.030-3.646), although values distribution was significantly less skewed among CF strains compared to non-CF and ENV strains (coefficient of variation: 70.0 vs 90.2, and 85.8%, respectively; *p *< 0.001). Similarly, among ENV strains variability in biofilm levels formed at 25°C was significantly lower than that observed at 37°C (36. 8 vs 85.8%, respectively; *p *< 0.001).

**Figure 3:**
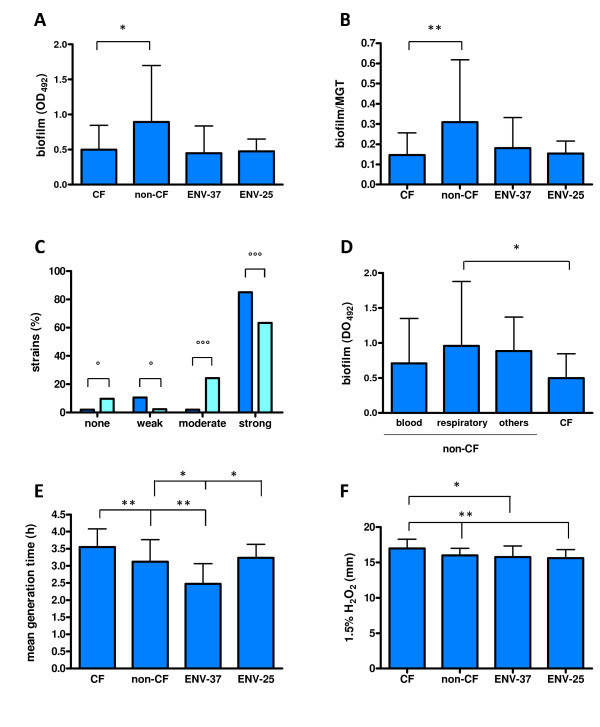
**Biofilm formation on polystyrene, growth rate, and susceptibility to oxidative stress among 98 clinical and environmental *S. maltophilia *strains**. **A**. Biofilm levels (mean + SD) formed by CF, non-CF, and ENV (ENV-37: 37°C-grown strains; ENV-25: 25°C-grown strains) isolates. **B**. Biofilm formation normalized on mean generation time (MGT) by CF, non-CF, ENV-37, and ENV-25 isolates. **C**. Percentage distribution of non-CF (blue bars) and CF (cyan bars) isolates belonging to no (OD_492 _≤ 0.096; n = 5), weak (0.096 < OD_492 _≤ 0.192; n = 6), moderate (0.192 < OD_492 _≤ 0.384; n = 11), or strong (OD_492 _> 0.384; n = 66) biofilm producer group. **D**. Biofilm formation (mean + SD) observed in non-CF strains, stratified by the isolation site, and CF strains. **E**. Mean generation time (mean + SD) of CF, non-CF, ENV-37, and ENV-25 isolates. **F**. Sensitivity to oxidative stress of CF, non-CF, ENV-37, and ENV-25 isolates. Results are expressed as mean (+ SD) diameter of inhibition zone formed by each isolate following exposure to 1.5% (vol/vol) H_2_O_2_. * *p *< 0.05 or ** *p *< 0.01, ANOVA followed by Bonferroni's multiple comparison post-test. ° *p *< 0.05 or °°° *p *< 0.0001, Fisher's exact test.

The mean biofilm formed by CF strains as a whole was significantly lower than that formed by non-CF strains (OD_492_, mean ± SD: 0.498 ± 0.348 vs 0.893 ± 0.806, respectively; *p *< 0.05) (Figure 3A), even after normalization on mean generation time (biofilm/MGT: 0.14 ± 0.11 vs 0.31 ± 0.31; CF vs non -CF strains, respectively; *p *< 0.01) (Figure 3B). No difference in biofilm formation was observed between clinical and ENV isolates (Figure 3A). With regard to biofilm categories, a significantly higher percentage of weak and strong biofilm producers was found in non-CF strains compared to CF ones (weak: 10.6 vs 2.4%, respectively, *p *< 0.05; strong: 85.1 vs 63.4%, respectively, *p *< 0.0001) (Figure 3C). Contrarily, CF group exhibited a significantly higher proportion of moderate biofilm forming strains (23.0 vs 2.0%, respectively, *p *< 0.0001) (Figure 3C). No significant difference in biofilm levels formed by non-CF strains was found according to the isolation site, although among respiratory strains, non-CF strains produced significantly higher biofilm levels compared to CF ones (0.960 ± 0.919 vs 0.498 ± 0.348, respectively; *p *< 0.05) (Figure 3D).

### CF isolates grow slower and are more sensitive to H_2_O_2_, compared to non-CF ones

CF isolates showed higher mean generation time compared to non-CF ones (3.5 ± 0.5 h vs 3.1 ± 0.6 h, respectively; *p *< 0.001) (Figure 3E). Indeed, ENV isolates grown at 37°C exhibited a significantly lower generation time compared to that observed at 25°C (2.5 ± 0.6 h vs 3.2 ± 0.4 h, respectively; *p *< 0.05) (Figure 3E). No significant relationship was found between growth rate and the biofilm biomass formed, regardless of group considered (data not shown).

Susceptibility to oxidative stress was evaluated by measuring the zone of inhibition formed by each strain following exposure to 1.5% H_2_O_2_. The mean zone of inhibition exhibited by CF strains (17.0 ± 1.3 mm) resulted to be significantly higher than that observed by non-CF (16.0 ± 1.0 mm; *p *< 0.01), and ENV strains (15.6 ± 1.2, and 15.8 ± 1.6 mm, for ENV-25, and ENV-37, respectively; *p *< 0.05) (Figure 3F).

### Phenotypic characteristics exhibited by CF sequential isogenic isolates undergo alterations during the course of chronic infection

**Figure 4:**
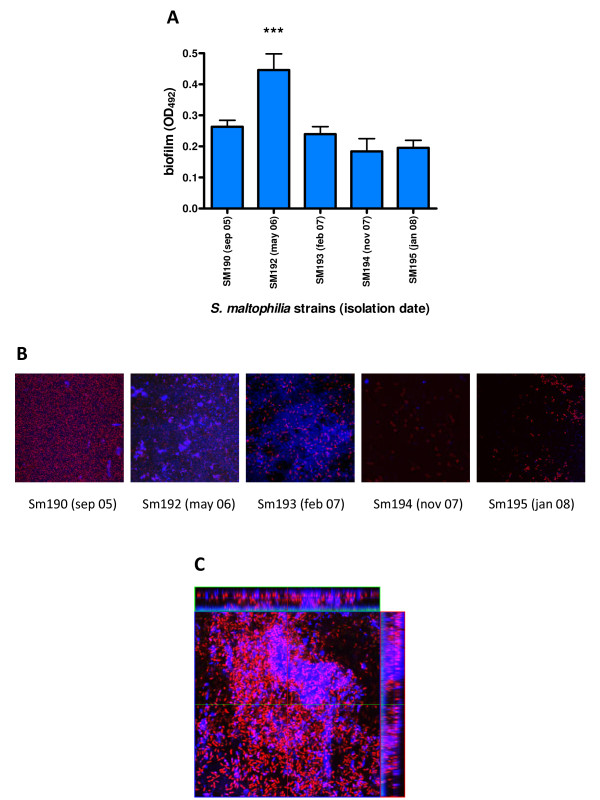
**Biofilm formed by *S. maltophilia *sequential strains isolated from the same CF patient**. **A**. Biofilm formation on polystyrene, assessed by microplate colorimetric assay. PFGE analysis revealed that all strains belonged to the same pulsotypes 23.1. *** *p *< 0.001, Sm192 vs other strains, ANOVA-test + Bonferroni's multiple comparison test. **B**. CLSM examination of biofilm formed by sequential isolates belonging to pulsotype 23.1 after 24 h of development. **C**. CLSM examination of *S. maltophilia *Sm192 biofilm after 24 h of development. Orthogonal images, collected within the biofilm as indicated by the green and red lines in the top view, showed that biofilm consisted of cells forming a multilayered structure (red, propidium iodide-stained) embedded in an abundant extracellular polymeric substance (blue, concanavalin A-stained). Image capture was set for simultaneous visualization of both red and blue fluorescence. Magnification, ×100.

Five *S. maltophilia *strains, isolated from the same CF patient over a period of 3 years and belonging to the same pulsotype, were investigated for phenotypic variations with regard to biofilm formation, mean generation time, swimming and twitching motility, and susceptibility to H_2_O_2_. As shown in Figure 4A, biofilm amount formed by Sm192 (strong biofilm producer) was significantly (*p *< 0.001) higher than other genetically indistinguishable isolates (moderate biofilm producers). Spectrophotometric results were confirmed by Confocal Laser Scanning Microscopy (CLSM) analysis showing significant differences in biofilm ultrastructure formed by the sequential isolates (Figures 4B-C). In particular, the biofilm formed by Sm192 strain resulting to be the most complex, revealing a multilayered cell structure (64-70 μm, depth) embedded in an abundant extracellular polymeric substance (EPS) (Figure 4C). These features were not observed for the other isolates showing either poor attachment (strains Sm194 and Sm195) or forming monolayer biofilm lacking EPS (strain Sm190) (Figure 4B).

Significant differences were also found among sequential isolates in some cases concerning susceptibility to oxidative stress (Sm194 vs Sm190, *p *< 0.05; Sm194 vs Sm192, *p *< 0.001) and swimming motility (Sm193 vs Sm194 and Sm195, *p *< 0.001) (data not shown).

### Swimming and twitching motilities are critical for biofilm development in CF strains

Overall, 9 nonmotile strains, 4 non-CF strains and 5 CF strains, with neither swimming nor twitching motility were observed, with only 2 of them resulting in the inability to form biofilm. No significant differences were seen in motility, in the percentage of motile strains, and in the mean motility level between CF and non-CF isolates (data not shown). Similarly, among ENV isolates growth temperature did not significantly affect neither swimming nor twitching motility (data not shown).

Interestingly, swimming and twitching motilities were positively correlated to biofilm biomass (Pearson r: 0.528 and 0.625, respectively; *p *< 0.0001) in CF strains only. No statistically significant differences were found among the motility patterns (swimming+/twitching+, swimming+/twitching-, swimming-/twitching+, and swimming-/twitching-) with respect to the biofilm formed (data not shown).

### CF and non-CF isolates show comparable virulence in a mouse model of lung infection

**Figure 5:**
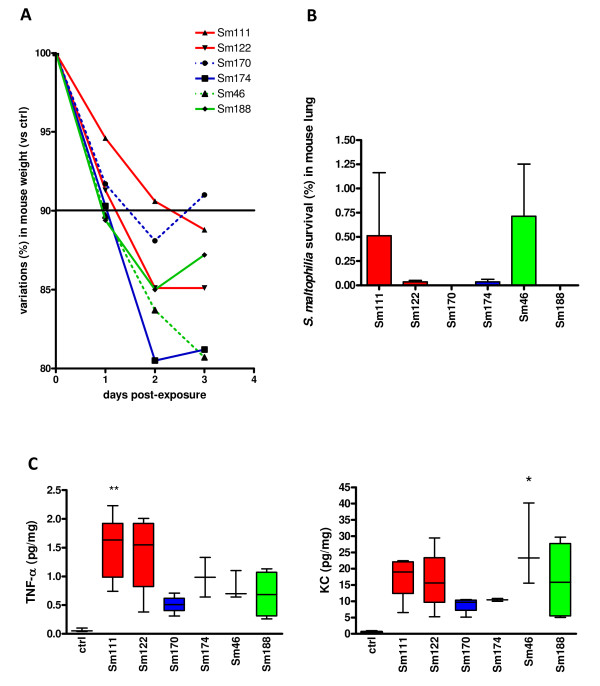
**Mouse model of acute lung infection by C F and non-CF *S. maltophilia *strains**. DBA/2 mice (n = 8, for each strain) were exposed on day 0 to aerosolized CF (Sm111 and Sm122 strains, from respiratory specimens) or non-CF (Sm170 and Sm174 strains, from respiratory specimens; Sm46 and Sm188 strains, from blood) *S. maltophilia *in PBS. Control mice were exposed to aerosolized PBS only. **A**. Weight monitoring during *S. maltophilia *lung infection. Results are expressed as percentage of weight loss with respect to control mice (100%). The horizontal line shows a 10% weight loss with regard to mean body weight of control mice. Differences in weight reduction were all significant (*p *< 0.01, Fisher's exact test) compared to control mice, except for Sm111 exposed mice at day 1 post-exposure (p.e.). **B**. *S. maltophilia *survival in mouse lungs 3 days p.e.. For each exposure, four mice each were included for determination of bacterial deposition to the lungs at 1 h and 3 days p.e.. Results are expressed as mean + SD. **C**. Cytokine levels measured on day 3 p.e. in lung homogenates. Results were normalized to the lung wet weight (pg/mg) and expressed as box and whiskers: the box extends from the 25^th ^percentile to 75^th ^percentile, with a line at the median (50^th ^percentile); the whiskers indicate the lowest and the highest value. * *p *< 0.05 or ** *p *< 0.01, Kruskal-Wallis test followed by Dunn's multiple comparison post-test.

As shown in Figure 5A, a weight reduction of at least 10% was observed on day 1 post-exposure (p.e.) in mice infected with invasive Sm46 and Sm188 strains and those exposed to non-CF Sm174, and later for mice exposed to CF strains (on day 2 and 3 p.e. for Sm122 and Sm111 strains, respectively). By day 1 p.e. the mean weight of infected mice was significantly (*p *< 0.01) lower than that of control mice. By day 2 p.e., only infected mice with non-CF strains (Sm174, Sm170) and the invasive Sm188 strain slowly started regaining weight, although only mice infected with Sm170 strain regained it completely on day 3 p.e.. Control mice lost not more than 1% of their body weight during the study-period monitored. All infected mice showed symptoms of slow responsiveness and piloerection from day 1 through day 3 p.e..

Lung clearance results of *S. maltophilia *infection are summarized in Figure 5B. The initial deposition of *S. maltophilia *in the mouse lung was assessed by viable count 1 h p.e.. All *S. maltophilia *strains were almost completely eradicated from mouse lung (> 99%), while Sm111 CF and Sm46 non-CF blood isolates were eradicated less effectively (0.51 and 0.71% retention, respectively) than non-CF respiratory strains (0.04% retention), although these differences were not statistically significant. No correlation was found between in vitro biofilm formation and in vivo lung colonization.

Pulmonary levels of cytokines detected on day 3 p.e. are shown in Figure 5C. Higher levels of TNF-α were significantly observed in the lungs of mice infected by Sm111 CF strain, compared to control mice (median: 1.63 vs 0.050 pg/mg, respectively; *p *< 0.01). Moreover, higher levels of KC were observed on day 3 p.e. in the lungs of mice infected by invasive Sm46 strain, compared to control mice (median: 23.28 vs 0.42 pg/mg, respectively; *p *< 0.01).

### Different genotypes are associated to strong biofilm formation in CF and non-CF isolates

PCR-based typing of 89 (84 clinical, 5 ENV) *S. maltophilia *strains for *spgM*, *rmlA*, and *rpfF *genes showed an overall prevalence of 88.8, 65.2, and 61.8%, respectively.

The presence of *rmlA, spgM *or *rpfF *did not significantly affect the mean amount of biofilm formed by CF or non-CF isolates. However, considering the strain population as a whole, the presence of *rmlA *significantly improved biofilm formation (0.820 ± 0.785 vs 0.415 ± 0.278, *rmlA^+ ^*vs *rmlA*^-^, respectively; *p *= 0.01).

With regard to biofilm categories, in CF strains displaying strong and moderate biofilm-producer phenotype the frequencies of *spgM*^+ ^and *rpfF*^+ ^isolates were significantly (*p *< 0.01) higher than *rmlA*^+ ^ones (strong biofilm producer: 92.3 vs 84.6 vs 61.5%, respectively; moderate biofilm producers: 90 vs 60 vs 20%, respectively). Among non-CF strong biofilm producer strains, frequencies of *spgM*^+ ^and *rmlA*^+ ^strains were significantly (*p *< 0.01) higher than *rpfF*^+ ^ones (88.8 vs 83.3 vs 55.5%, respectively).

Eight genotypes were observed with wide range percentages (from 1.1 to 34.8%) and those with the highest frequency were *rmlA*^+^/*spgM*^+^/*rpfF*^+ ^(34.8%), *rmlA*^-^/*spgM*^+^/*rpfF*^+ ^(23.6%), and *rmlA*^+^/*spgM*^+^/*rpfF*^- ^(21.3%).

**Figure 6:**
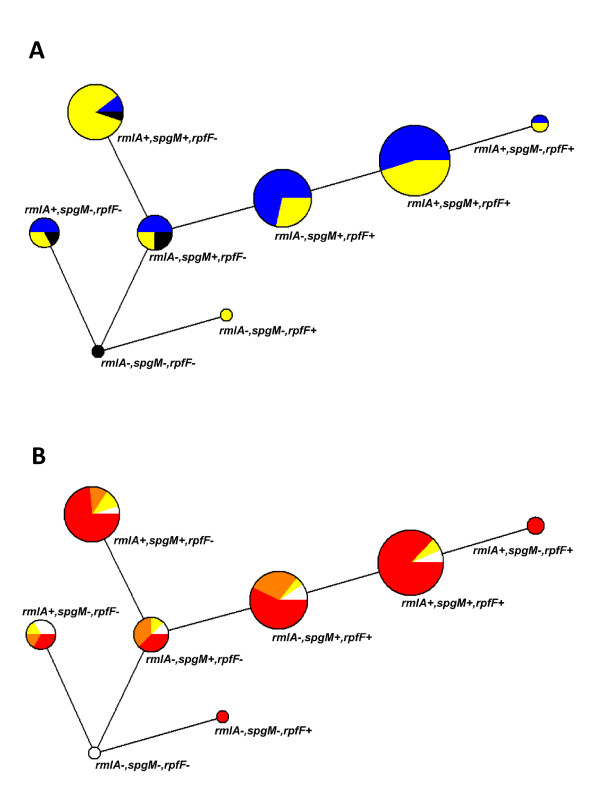
**Proportion of *S. maltophilia *genotypes and association with biofilm formation**. **A**. Genetic network representing proportion of genotypes found in CF (blue), non-CF (yellow), and ENV (black) strain population. *rmlA*^-^/*spgM*^+^/*rpfF*^+ ^genotype was statistically more represented in CF than non-CF group (71.4 vs 28.6%, respectively; *p*<0.0001, AMOVA); *rmlA*^+^/*spgM*^+^/*rpfF*^- ^genotype was statistically more represented in non-CF than CF group (84.2 vs 10.5%, respectively; *p *< 0.0001, AMOVA). **B**. Genetic network representing association between genotypes and biofilm formation (red: strong biofilm producers; orange: moderate biofilm producers; yellow: weak biofilm producers; white: no biofilm producers). *rmlA*^-^/*spgM*^+^/*rpfF*^+ ^and *rmlA*^+^/*spgM*^+^/*rpfF*^- ^genotypes were statistically associated to strong biofilm producers (Pearson r: 0.82 and 0.88, respectively; *p *< 0.01).

Analysis of molecular variance (AMOVA) followed by Pairwise Fst values comparison highlighted significant variance (*p *< 0.01) in genotypes distribution between CF and non-CF strains, and also between ENV and respectively CF and non-CF strains. In particular, *rmlA*^-^/*spgM*^+^/*rpfF*^+ ^and *rmlA*^+^/*spgM*^+^/*rpfF*^- ^genotypes were differentially observed, the first one accounting for 71.4% and 28.6% (*p *< 0.0001) while the second one for 10.5% and 84.2% (*p *< 0.0001) in CF and non-CF strains, respectively (Figure 6A).

Within each group the genotypes did not significantly differ for mean amount of biofilm formed (data not shown). However, with regard to genotype *rmlA*^+^/*spgM*^+^/*rpfF*^+ ^CF isolates formed significantly decreased biofilm amounts compared to non-CF ones (0.556 ± 0.485 vs 1.110 ± 0.832, respectively; *p *< 0.05).

The genetic network in Figure 6B shows the proportion of strong-, moderate-, weak- and no-biofilm producer strains associated to each observed genotype. Correlation analysis showed that genotypes differentially detected in CF (*rmlA*^-^/*spgM*^+^/*rpfF*^+^) and non-CF (*rmlA*^+^/*spgM*^+^/*rpfF*^-^) strains were both associated to strong biofilm producers (Pearson r: 0.82, and 0.88 for CF and non-CF strains, respectively; *p *< 0.01). However, CF genotypes were also correlated to no biofilm producer strains (Pearson r = 0.72, *p *= 0.02) while non-CF strains were correlated to weak biofilm producer ones (Pearson r = 0.93, *p *< 0.0001).

## Discussion

In the present study, we comparatively studied phenotypic and genotypic traits of 98 *S. maltophilia *isolates (41 CF, 47 non-CF, and 10 ENV strains) collected from geographically diversified areas.

To date, the epidemiology of *S. maltophilia *in CF patients has not been fully clarified. Molecular typing methods revealed a genetically high diversity within *S. maltophilia *strains, both from hospitalized CF and non-CF patients [21-32]. Our results confirmed the high degree of diversity between isolates from hospitalized CF and non-CF patients, thus suggesting that CF pulmonary *S. maltophilia *infections are mainly associated with a predominant strain. Nevertheless, we observed several examples of PFGE types shared by multiple isolates in both CF (pulsotypes 23.1 and 24.1) and non-CF (pulsotypes 1.1, 2.1, and 3.1) patients. In particular, the major PFGE type 23 clone identified, represented by 4 strains recovered from non replicate CF patients, likely indicate the occurrence of person-to-person transmission of *S. maltophilia *strains, the acquisition of this specific clone from a common source, or an independent acquisition of a widely-spread strain type. The dissemination and spread of a specific clone may be due to the circulation of a transmissible strain among CF patients, probably due to a better fitness of this specific clone in the CF pulmonary niche or from an environmental source.

Interestingly, distinct PFGE types were found between CF isolates and non-CF isolates. Further studies are warranted to evaluate if factors associated to the virulence could affect this important segregation among these two settings.

These results could reflect an extensive spread of *S. maltophilia *in the environment thus suggesting the existence of natural reservoirs of bacterial strains able to cause pathogenicity once acquired by CF patients. Contrary to *P. aeruginosa*, it has not been reported yet that *S. maltophilia *is capable of making the transition from an environmental state to a colonizing state in CF patients. However, Marzuillo et al [33] found a persistence of the same *S. maltophilia *strain in water, taps, and sinks of different rooms of an Italian CF center, although no correlation was observed between clinical and water-associated isolates. Furthermore, we recently observed that environmental *S. maltophilia *is potentially virulent, although to a lesser extent than CF one, in a murine model of lung infection [34]. Moreover, our results showed that two environmental isolates (C34, A33) shared genetically related PFGE type with a non-CF isolate (Sm184). Thus, it is plausible to hypothesize that the acquisition of pathogenic *S. maltophilia *strains can occur directly from the natural environment.

*S. maltophilia *is capable of adhering to and forming biofilm not only on polystyrene [12-14,16,35], but also on CF bronchial epithelial cells [17], suggesting that biofilm formation could be a critical step in colonisation of CF lung.

While *S. maltophilia *possesses complex, diversified genomes [1] and forms biofilms, it is not yet known whether there are any variations in biofilm formation among clonally diverse clinical and environmental isolates. In the present study, microtiter colorimetric assay for biofilm formation showed a wide range of biofilm formation ability in both CF and non-CF groups, from biofilm-deficient phenotypes to those producing structurally complex biofilms. However, when grouped, CF isolates were found to form an amount of biofilm significantly lower compared to that observed among non-CF isolates. To exclude the possibility that these differences in biofilm formation could arise from differences in growth efficiency [36], biofilm levels were normalized on growth rate calculated for each strain.

Although the mean growth rate of CF isolates was significantly lower than non-CF ones - probably because of the phenotypic regulation of virulence factor expression by quorum sensing mechanisms or by in vivo bacterial microevolution driven by selective lung environmental conditions, mechanisms already described in bacteria [37-39] - significant differences in biofilm formation were maintained also after normalization, thus indicating that in *S. maltophilia *biofilm formation is not influenced by growth rate.

The reduced efficiency in forming biofilm and the increased mean generation time exhibited by CF isolates could be the consequences of *S. maltophilia *adaptation to a stressed environment such as CF lung [40-42]. To verify this hypothesis, five isogenic sequential *S. maltophilia *strains isolated from the same CF patient over a period of 3 years were investigated for phenotypic variations. Our results showed that isogenic serial strains significantly differ in biofilm forming ability, susceptibility to oxidative stress, and swimming motility suggesting that different *S. maltophilia *phenotypes evolve within the CF respiratory tract during chronic infection. Particularly, the reduction in biofilm formation ability of sequential isolates is suggestive for the phenotypic conversion of *S. maltophilia *during chronic infection. CLSM analysis showed that isolates from the early periods of chronic infection were able to form uniform flat biofilms or highly structured, multilayered and exopolysaccharide matrix-encased, biofilms. On the contrary, isolates recovered from the late phase of chronic infection showed a significant reduction in adherence, lacking ability to form a mature biofilm. Significant differences were also found with regard to susceptibility to oxidative stress and swimming motility.

These results suggest that the onset of chronic infection could be transformative for *S. maltophilia*, probably reflecting an adaptive behavior that enables *S. maltophilia *to survive to the environmental stresses that are likely to be encountered within the habitat of the CF lung, such as (oxidative stress) low free iron, and anaerobic conditions [43]. In support of this, the phenotypic changes observed in *P. aeruginosa *isolates collected during different periods of chronic infection from CF patients, included loss of flagella or pilus mediated motility, loss of O antigen components of the LPS, as well as appearance of auxotrophic variants [39,41,44].

Biofilm formation is a multistep process requiring participation of structural appendages, such as flagella and type IV pili [44-47]. Recently, we showed that the flagellum plays a direct role, as an adhesin, in *S. maltophilia *adhesion to IB3-1 bronchial cells [17].

To test whether variations in biofilm formation we observed in *S. maltophilia *could be due to altered activities of these structural appendages, we measured the swimming and twitching abilities of the tested isolates. Although most of the isolates tested were able to move by swimming and twitching motilities, a lack of both motilities was observed in 4 (8.5%) non-CF strains and 5 (12.2%) CF strains. Of these 9 non-motile strains, only 2 CF strains were unable to form biofilm, thus suggesting that in *S. maltophilia*, as well as *P. aeruginosa *[48], motility is not an absolute requirement for biofilm formation [48]. It is worthy of note that both swimming and twitching motilities were positively correlated with biofilm levels in CF group only. Taken together, our observations indicate that, although not involved in the initial attachment of *S. maltophilia*, flagella and type IV pili play a critical role in biofilm development in the CF isolates, thus suggesting the existence of a peculiar mechanism involved in the control of biofilm formation in the CF lung.

The molecular mechanisms of biofilm formation have not been extensively studied in *S. maltophilia*. Recently, Fouhy et al. [18] described in *S. maltophilia *a cell-cell signaling mediated by a diffusible signal factor (DSF, *cis*-11-methyl-2-dodecenoic acid) whose synthesis is fully dependent on rpfF. The *rpfF *mutant showed severely reduced motility, altered LPS profiles and decreased biofilm formation [18]. Huang et al. [19] found that alteration in lipopolysaccharide (LPS), caused by the *rmlA *mutation, contributed to changes in flagella and type IV pili, thus interfering with motility, attachment, and biofilm formation [19]. A bifunctional *spgM*-encoded enzyme with both phosphoglucomutase (PGM) and phosphomannomutase activities was also found in *S. maltophilia *[20]. Since *spgM *gene is a homologue of the *algC *gene, responsible for the production of a PGM associated with LPS and alginate biosynthesis in *P. aeruginosa*, it is plausible to hypothesize an involvement of this gene also in *S. maltophilia *biofilm formation.

In the present study we also focused our efforts on the relationship between biofilm formation and the presence of *rpfF*, *rmlA *and *spgM *genes. Our results showed that *rmlA*^-^/*spgM*^+^/*rpfF*^+ ^and *rmlA*^+^/*spgM*^+^/*rpfF*^- ^genotypes are significantly associated to CF and non-CF groups, respectively. Furthermore, we found a significant association between the detection of these genes and the biofilm expression profiles, indicating that strong biofilm-producer isolates are significantly associated to both genotypes.

Overall, our results may endorse the central role of *spgM *gene in *S. maltophilia *biofilm formation ability, whose presence is significantly associated to a strong biofilm formation, both in non-CF and CF strains. On the contrary, the contribution of *rpfF *and *rmlA *is different on the basis of the group considered, thus confirming that biofilm formation is differently regulated in CF and non-CF strains.

The hallmark of the infected CF lung is a chronic neutrophil-dominated airway inflammation, and cytokine release [15,49]. Activated neutrophils and macrophages are major sources of oxygen free radicals including hydrogen peroxide. Jobsis et al. [50] recently showed that in CF children with acute infective pulmonary exacerbations exhaled H_2_O_2 _levels were higher than those found in healthy children.

Starting from these evidences we evaluated *S. maltophilia *sensitivity to oxidative stress by exposure to H_2_O_2 _on solid agar. Our results revealed that CF isolates exhibited a higher level of susceptibility than the non-CF strains to this particular ROI species. As already stated by Head & Yu [51] with regard to *P. aeruginosa *CF isolates, it could also be possible in *S. maltophilia *CF isolates an impaired production of superoxide dismutase, catalase or peroxidase, thus explaining their limited ability to survive and proliferate under in vitro oxidative stress.

The virulence of *S. maltophilia *from different sources was evaluated by using an aerogenic acute lung infection mouse model we recently described [15]. Although pulmonary eradication on day 3 p.e. resulted high (> 99%) for all strains tested, Sm111 CF and Sm46 non-CF blood isolates were markedly less capable of being cleared than non-CF respiratory ones. The apparent disagreement between these findings and the higher susceptibility to H_2_O_2 _exhibited by CF isolates is probably due to the fact that neutrophil migration from the bloodstream to the lungs occurs in the early hours following infection. No correlation was found between in vitro biofilm formation and in vivo lung colonization, reasonably because the aerosol mouse model we used simulates an acute infection condition caused by planktonic cells, thus not allowing biofilm formation. Contrary to the findings by Waters et al [4], our results suggested that *S. maltophilia *CF strains were more immunostimulatory than non-CF ones with regard to TNF-α - a potent proinflammatory cytokine that induces neutrophil and macrophage activation - and KC - a keratinocyte-derived chemoattractant for neutrophils. This is a very important feature in the initial colonization of the airways and development of pneumonia. Further in vivo studies employing an adequate number of isolates are needed to clarify the clinical significance of our results.

## Conclusions

Our results showed that *S. maltophilia *CF strains significantly differ from non-CF ones in some phenotypic traits. Considering that adaptability is the key to successful colonization of an environmental niche, these particular responses taken characteristically by CF isolates could be the biological price to evade the hostile and heterogeneous CF lung environments. In fact, the CF lung environment, representing a more extreme environment to adapt to than other clinical ones, could tax the cellular resources of CF strains to a greater extent than those of the non-CF clinical isolates, thus resulting in the selection of a "CF phenotype" for *S. maltophilia*. The elucidation of molecular mechanisms underlying these phenotypic differences might be relevant to the identification of new targets for designing rational and effective methods to combat and eradicate *S. maltophilia *infection.

## Methods

### Bacterial isolates and growth conditions

Overall, 98 *S. maltophilia *isolates were investigated: 41 strains collected from the sputa of CF patients attending the CF Unit at "Bambino Gesù" Children's Hospital and Research Institute of Rome; 47 strains collected from different sites (30 from respiratory tract, 10 from blood, and 7 from swabs) in non-CF patients attending "Bambino Gesù" Children Hospital of Rome, or "Spirito Santo" Hospital of Pe scara; and 10 strains (ENV) isolated in Czech Republic from several environmental sources (paddy, soil, rhizosphere tuberous roots, and waste water). Since in severely ill chronic obstructive pulmonary disease (COPD) patients *P. aeruginosa *clones similar to those in CF persists [52], patients with COPD were not enrolled in the present study. All clinical isolates represented non-consecutive strains isolated from different patients, except for 2 CF patients with 7 and 3 isolates, respectively. The isolates were identified as *S. maltophilia *by biochemical tests using manual (API 20-NE System; BioMérieux, Marcy-L'Etoile, France) or automated (Vitek; BioMérieux) systems, then stored at -80°C until use when they were grown at 37°C (and also at 25°C, in the case of ENV strains) in Trypticase Soy broth (TSB; Oxoid SpA; Garbagnate M.se, Milan, Italy) or Mueller-Hinton agar (MHA; Oxoid) plates unless otherwise noted.

### Genetic relatedness by PFGE and cluster analysis

After digestion of DNA with the restriction enzyme *XbaI *as previously described [24,27,28], PFGE was carried out as follows: initial switch time and final switch time were 5 and 35 sec, respectively; DNA fragments were run with a temperature of 12°C for 20 h at 6.0 V/cm with an included angle of 120°. Isolates with identical PFGE patterns were assigned to the same PFGE type and subtype. Isolates differing by one to three bands were assigned to different PFGE subtypes but to the same PFGE type and were considered genetically related. Isolates with PFGE patterns differing by more than 4 bands were considered genetically unrelated and were assigned to different PFGE types. PFGE types were analyzed with BioNumerics software for Windows (version 2.5; Applied Maths, Ghent, Belgium). The DNA banding patterns were normalized with bacteriophage lambda concatemer ladder standards. Comparison of the banding patterns was performed by the UPGMA and with the Dice similarity coefficient. A tolerance of 1.5% in band position was applied during DNA patterns comparison.

### Biofilm formation assay

Overnight cultures in TSB were corrected with fresh TSB to an OD_550 _of 1.00 (corresponding to about 1 × 10^9 ^CFU/ml). Two-hundred microliters of 1:100 diluted inoculum were dispensed to each well of a sterile flat-bottom polystyrene tissue culture 96-wells microtiter (Iwaki, Bibby srl; Milan, Italy) and incubated at 37°C for 24 h. Biofilm formation by ENV strains was also assessed at 25°C. Non-adherent cells were removed by being washed three times in sterile PBS (pH 7.3; Sigma-Aldrich Co; Milan, Italy), and biofilm biomass was then measured by crystal violet assay. Briefly, biofilm samples were fixed for 1 h at 60°C, stained for 5 min at RT with 200 μl Hucker-modified crystal violet, then rinsed in standing water and allowed to dry. Biofilm samples were estained with 250 μl of 33% glacial acetic acid for 15 min, and the optical density at 492 nm (OD_492_) was read. Considering a low cut-off (OD_c_) represented by 3×SD above the mean OD of control wells, strains were classified into the following categories: no biofilm producer (OD ≤ OD_c_), weak biofilm producer (OD_c _< OD ≤ 2 × OD_c_), moderate biofilm producer (2 × OD_c _< OD ≤ 4 × OD_c_), and strong biofilm producer (4 × OD_c _< OD) [53].

### Measurement of growth rate

Two-hundred microliters of the 1:100 diluted standardized inoculum were dispensed in each well of a microtiter plate, and OD_570 _readings were taken every 15 min for a total time of 15 h by a microplate reader (SpectraMax 190; Molecular Devices Inc.; Sunnyvale, CA, USA). Considering the exponential growth phase selected on a graph of ln OD_570 _versus time, mean generation time (MGT) was calculated as follows: MGT = ln2/μ, where μ (growth rate) = (lnOD _t _- lnOD_t0_)/t.

### Swimming and twitching motilities

Motility assays were performed according to the method described by Rashid et al. [54], with some modifications. i) Swimming assay: a single colony from an overnight MHA-growth was inoculated at the surface of swimming agar (10 g/liter tryptone, 5 g/liter NaCl, 3 g/liter agar); after inoculation, the plates were then wrapped to prevent dehydration and incubated at 37°C for 24 h, and results were expressed as diameter (mm) of growth zone. ii) Twitching motility: a single colony from an overnight MHA-growth was inoculated, by using an inoculation needle, to the bottom of the Petri dish plate containing twitching agar (1% TSB solidified with 1% agar); after incubation at 37°C for 72 h, agar was removed and the zone of motility at the agar/Petri dish interface was stained with crystal violet and measured in millimeters.

### Sensitivity to oxidative stress

Assays were carried out by a disk assay adapted by Hassett et al. [55]. Briefly, 100-μl aliquots from TSB cultures in mid-log or stationary phases of growth were uniformly spread on TSA plates containing 2% agar. Sterile filter paper 7-mm diameter disks (Oxoid) were placed on TSA surface, and the disks were spotted, in triplicate on each plate, with 10 μl of 1.5% H_2_O_2_. The diameter of the zone of growth inhibition around each disk was measured after 24 h of incubation at 37°C.

### CLSM

Biofilm samples, prepared as stated above, were fixed in formaldehyde-paraformaldehyde, and stained with propidium iodide (PI; Molecular Probes Inc.; Eugene, OR, USA) and concanavalin A (ConA, Alexa Fluor 647 conjugate; Molecular Probes Inc.). CLSM analysis was performed with an LSM 510 META laser scanning microscope attached to an Axioplan II microscope (Carl Zeiss SpA; Arese, Milan, Italy). The excitation wavelengths were 458 [Argon laser], and 543 nm [He-Ne laser], and emission wavelengths were 488, and 615 nm for PI and ConA, respectively. Depth measurements were taken at regular intervals across the width of the device. To determine the structure of the biofilms, a series of horizontal (*x-y*) optical sections were taken throughout the full length of the biofilm. Confocal images of blue (ConA) and red (PI) fluorescence were conceived simultaneously using a track mode. Images were captured and processed for display using Adobe Photoshop (Adobe Systems Italia, Rome, Italy) software.

### PCR-based genotyping for *rmlA*, *spgM*, and *rpfF*

Bacterial DNA was isolated by using the High Pure PCR Template Preparation Kit (Roche Diagnostics S.p.A, Milan, Italy). Purified DNA was amplified and visualized on 2% agarose gel. PCR oligonucleotides were respectively 5'- GCAAGGTCATCGACCTGG-3' and 5'-TTGCCGTCGTAGAAGTACAGG-3' (82 bp) for *rmlA*, 5'-GCTTCATCGAGGGCTACTACC-3' and 5'-ATGCACGATCTTGCCGC-3' (80 bp) for *spgM *and, finally, 5'-CTGGTCGACATCGTGGTG-3' and 5'-TGATCCGCATCATTTCATGC-3' (151 bp) for *rpfF*. All PCRs were carried out in 30 μl volumes with 10 mM Tris (pH 8.3), 2.5 mM MgCl_2_, 200 mM dNTP, 1.25 U of *Taq*-pol (EuroClone S.p.A., Milan, Italy), 0.5 μM of each pr imer, and 3 μl of DNA extract. Amplification conditions were as follows: 30 cycles of 60°C for 20 sec, 72°C for 30 sec, and 94°C for 20 sec. To verify the specificity of the amplification test a pool of 21 PCR products was directly sequenced using the ABI Prism RR Big-Dye Terminator Cycle Sequencing Kit on an ABI Prism 310 Genetic Analyzer (Applied Biosystems).

### *S. maltophilia *aerosol infection mouse model

The virulence of selected strains from diverse clinical settings - including CF (no biofilm producer Sm111 strain, and strong biofilm producer Sm122 strain) and non-CF (strong biofilm producer Sm170 and Sm174 strains) respiratory specimens, as well as blood specimens (strong biofilm producer Sm46 and Sm188 strains) - was comparatively evaluated by using an aerogenic infection mouse model [15]. All procedures involving mice were reviewed and approved by the Animal Care and Use Committee of "G. d'Annunzio" University of Chieti-Pescara. Eight DBA-2 inbred, specific pathogen-free mice (Charles River Laboratories Italia srl, Calco, Italy) were exposed for 60 min to the nebulisation of a standardized bacterial suspension (1.6 × 10^11 ^CFU/ml) prepared in PBS (Sigma-Aldrich). In each group, four mice were sacrificed by carbon dioxide at t = 1 h and t = 3 days post-exposure. For quantitative bacteriology analysis, 10-fold dilution series of homogenized lungs were plated on MHA for counting. For cytokine measurements, a protease inhibitors cocktail (Protease Inhibitor Cocktail kit; Pierce, Rockford, IL, USA) was added to the lung samples immediately after collection. Lung homogenates were centrifuged (1,500 × g, 4°C, 10 min), then the supernatants were assayed for TNF-α and KC (Keratinocyte-derived Cytokine) levels by a multiplexing sandwich-ELISA system based on chemiluminescent detection (SearchLight Chemiluminescent Array Kits; Endogen, Rockford, IL, USA), according to the manufacturer's recommendations. The detection limit for TNF-α and KC was 12.5 pg/ml and 6.0 pg/ml, respectively. The number of colonies for each lung and cytokine levels were normalized according to the wet weight of lung tissue, and showed as CFU/mg or pg/mg lung tissue, respectively.

### Statistical analysis

All experiments were performed at least in triplicate and repeated on two different occasions. Statistical analysis of results was conducted with GraphPad Prism version 4.00 (GraphPad software Inc.; San Diego, CA, USA), considering as statistically significant a *p *value < 0.05. Parametric (ANOVA-test followed by Bonferroni's multiple comparison test) or non-parametric (Kruskal-Wallis test followed by Dunn's multiple comparison test) tests were performed when data were normally distributed or not, respectively. Differences between frequencies were assessed by Fisher's exact test. The Pearson's correlation coefficient was calculated to determine the association between two variables. Analysis of Molecular Variance (AMOVA), as implemented in the Arlequin 2005 software [56], was performed to analyze frequencies of genotypes based on *rmlA, spgM*, and *rpfF *detection. For all calculations, significance was assessed by 1,000 permutations. The F-statistic (Fst) approach [57] was applied to verify statistical differences in genotype distributions among *S. maltophilia *CF, non-CF and environmental strains. Genetic networks were generated using the median-joining algorithm implemented in NETWORK 4.516 software (Fluxus Technology Ltd).

## Competing interests

The authors declare that they have no competing interests.

## Authors' contributions

AP, SP, and VC performed biofilm formation, growth rate, motility, sensitivity to oxidative stress, confocal microscopy, and in vivo assays. AP also drafted the manuscript. FV took care of PCR-based genotyping. GG and GD carried out pulsed-field gel electrophoresis and cluster analysis. EF, VS, and DD contributed by giving a medical point of view to the discussion of the results. EF also collected clinical strains used in the present work. GDB performed statistical analysis, and was involved in the design and coordination of the study, contributed to the revision of the manuscript, and gave their final approval of the version to be published. All authors read and approved the final manuscript.
